# Technical Metrics Used to Evaluate Health Care Chatbots: Scoping Review

**DOI:** 10.2196/18301

**Published:** 2020-06-05

**Authors:** Alaa Abd-Alrazaq, Zeineb Safi, Mohannad Alajlani, Jim Warren, Mowafa Househ, Kerstin Denecke

**Affiliations:** 1 College of Science and Engineering Hamad Bin Khalifa University Doha Qatar; 2 Institute of Digital Healthcare University of Warwick Coventry United Kingdom; 3 School of Computer Science University of Auckland Auckland New Zealand; 4 Institute for Medical Informatics Bern University of Applied Sciences Bern Switzerland

**Keywords:** chatbots, conversational agents, health care, evaluation, metrics

## Abstract

**Background:**

Dialog agents (chatbots) have a long history of application in health care, where they have been used for tasks such as supporting patient self-management and providing counseling. Their use is expected to grow with increasing demands on health systems and improving artificial intelligence (AI) capability. Approaches to the evaluation of health care chatbots, however, appear to be diverse and haphazard, resulting in a potential barrier to the advancement of the field.

**Objective:**

This study aims to identify the technical (nonclinical) metrics used by previous studies to evaluate health care chatbots.

**Methods:**

Studies were identified by searching 7 bibliographic databases (eg, MEDLINE and PsycINFO) in addition to conducting backward and forward reference list checking of the included studies and relevant reviews. The studies were independently selected by two reviewers who then extracted data from the included studies. Extracted data were synthesized narratively by grouping the identified metrics into categories based on the aspect of chatbots that the metrics evaluated.

**Results:**

Of the 1498 citations retrieved, 65 studies were included in this review. Chatbots were evaluated using 27 technical metrics, which were related to chatbots as a whole (eg, usability, classifier performance, speed), response generation (eg, comprehensibility, realism, repetitiveness), response understanding (eg, chatbot understanding as assessed by users, word error rate, concept error rate), and esthetics (eg, appearance of the virtual agent, background color, and content).

**Conclusions:**

The technical metrics of health chatbot studies were diverse, with survey designs and global usability metrics dominating. The lack of standardization and paucity of objective measures make it difficult to compare the performance of health chatbots and could inhibit advancement of the field. We suggest that researchers more frequently include metrics computed from conversation logs. In addition, we recommend the development of a framework of technical metrics with recommendations for specific circumstances for their inclusion in chatbot studies.

## Introduction

### Background

The potential of human-computer dialog to provide health care benefits has been perceived for many decades. In 1966, Weizenbaum’s ELIZA system caught the public imagination with its imitation of a psychotherapist through the relatively simple linguistic token manipulation possible at the time [1]. From the mid-1990s, a family of interventions based on automated telephone sessions (telephone-linked care) demonstrated effectiveness in promoting health adherence across a range of behaviors including medication, diet, and physical activity [2]. As mobile phones have become commonplace, a range of SMS text messaging–based interventions have been developed and trialed, with particular success in smoking cessation [3]. At the same time, internet/web-based interventions have shown the ability to promote positive health behavior change [4,5], and the interaction components associated with users sticking with an internet intervention are increasingly well understood and include the inclusion of dialog elements [6].

With the advent of smartphones, the distribution of highly interactive chatbots has been greatly facilitated, particularly with the ubiquitous use of app stores and wide installation of chat apps that can include chatbots, notably Facebook Messenger. Chatbots, as with other electronic health (eHealth) interventions, offer scalability and 24-hour availability to plug gaps in unmet health needs. For example, Woebot delivers cognitive behavior therapy and has been tested with students with depression [7]. The students who used Woebot significantly reduced their symptoms of depression over the study period as measured by the depression questionnaire PHQ-9, while those in the information control group (who instead read a self-help book) did not [7]. In recent years, artificial intelligence (AI) based on deep learning has created waves with its ability to outperform physicians at some diagnostic tasks [8,9]. XiaoIce is a social chatbot that emphasizes emotional connection and it has communicated with over 660 million active users since its launch in 2014 [10]; its performance shows that deep learning can be successfully applied to meaningful dialog tasks. Combining the factors of ease-of-distribution, successful applications, and AI methods to improve health chatbot performance, it is reasonable to expect health chatbots in increasing numbers and variety to take an increasingly serious role in the health care system.

### Research Problem and Aim

To be an evidence-based discipline requires measurement of performance. The impact of health chatbots on clinical outcomes is the ultimate measure of success. For example, did the condition (eg, depression, diabetes) improve to a statistically significant degree on an accepted measure (eg, PHQ-9 [11] or hemoglobin A1c [12], respectively), as compared to a control group? Such studies, however, may require large sample sizes to detect the effect and provide relatively little insight into the mechanism by which the chatbot achieves the change; additionally, studies may provide particularly little insight if the result is negative.

As an alternative and useful precursor to clinical outcome metrics, technical metrics concern the performance of the chatbot itself (eg, did participants feel that it was usable, give appropriate responses, and understand their input?). Appropriateness refers to the relevance of the provided information in addressing the problem prompted [13]. Furthermore, this includes more objective measures of the chatbot interaction, such as the number of conversational turns taken in a session or time taken, and measures that require some interpretation but are still well-defined, such as task completion. These technical measures offer a potential method for comparison of health chatbots and for understanding the use and performance of a chatbot to decide if it is working well enough to warrant the time and expense of a trial to measure clinical outcomes.

Previously, we had introduced a framework for evaluation measures of health chatbots to provide guidance to developers [14]. The framework development, however, was based on a relatively informal process vulnerable to the authors’ biases in terms of what studies were considered in its formulation. Therefore, the aim of this study is to use a rigorous review methodology to identify the technical metrics used by previous studies to evaluate health care chatbots. The final goal of these efforts is to be able to make recommendations for an evaluation framework for health chatbots.

## Methods

### Overview

To achieve the aforementioned objective, a scoping review was conducted. To conduct a transparent and replicable review, we followed the PRISMA (Preferred Reporting Items for Systematic Reviews and Meta-Analyses) Extension for Scoping Reviews (PRISMA-ScR) guidelines [15]. This research was conducted by an interdisciplinary team of researchers with backgrounds in nursing, computer science, and mental health applications.

### Search Strategy

#### Search Sources

For this review, we searched the following bibliographic databases November 1-3, 2019: MEDLINE (via EBSCO), EMBASE (Excerpta Medica Database; via Ovid), PsycINFO (via Ovid), CINAHL (Cumulative Index of Nursing and Allied Health Literature; via EBSCO), IEEE (Institute of Electrical and Electronics Engineers) Xplore, ACM (Association for Computing Machinery) Digital Library, and Google Scholar. We screened only the first 100 hits retrieved by searching Google Scholar, as it usually retrieves several thousand references ordered by their relevance to the search topic. We checked the reference list of the included studies to identify further studies relevant to the current review (ie, backward reference list checking). Additionally, we used the “cited by” function available in Google Scholar to find and screen studies that cited the included studies (ie, forward reference list checking).

#### Search Terms

The search terms were derived from previously published literature and the opinions of informatics experts. For health-related databases, we used search terms related to the intervention of interest (eg, chatbot, conversational agent, and chat-bot). In addition to intervention-related terms, we used terms related to the context (eg, health, disease, and medical) for non–health-related databases (eg, IEEE and ACM digital library). Multimedia Appendix 1 details the search strings used for searching each electronic database.

### Study Eligibility Criteria

The intervention of interest in this review was chatbots that are aimed at delivering health care services to patients. Chatbots implemented in stand-alone software or web-based platforms were included. However, we excluded chatbots operated by a human (Wizard-of-Oz) or integrated into robotics, serious games, SMS text messaging, or telephone systems. To be included, studies had to report a technical evaluation of a chatbot (eg, usability, classifier performance, and word error rate). We included peer-reviewed articles, dissertations, and conference proceedings, and we excluded reviews, proposals, editorials, and conference abstracts. This review included studies written in the English language only. No restrictions were considered regarding the study design, study setting, year of publication, and country of publication.

### Study Selection

Authors MA and ZS independently screened the titles and abstracts of all retrieved references and then independently read the full texts of included studies. Any disagreements between the two reviewers were resolved by AA. We assessed the intercoder agreement by calculating Cohen, which was 0.82 for screening titles and abstracts and 0.91 for reading full texts, indicating a very good agreement [16].

### Data Extraction

To conduct a reliable and accurate extraction of data from the included studies, a data extraction form was developed and piloted using 8 included studies (Multimedia Appendix 2). The data extraction process was independently conducted by two reviewers (MA and ZS) and a third reviewer (AA) resolved any disagreements. Intercoder agreement between the reviewers was good (Cohen κ=0.67).

### Data Synthesis

A narrative approach was used to synthesize the extracted data. After identifying all technical metrics used by the included studies to evaluate chatbots, we divided them into 4 categories based on the aspect of chatbots that the metrics evaluate. The 4 categories were formed after a discussion by the authors in which consensus was reached. For each metric, we identified how the studies measured it. Data synthesis was managed using Microsoft Excel (Microsoft Corporation).

## Results

### Search Results

**Figure 1:**
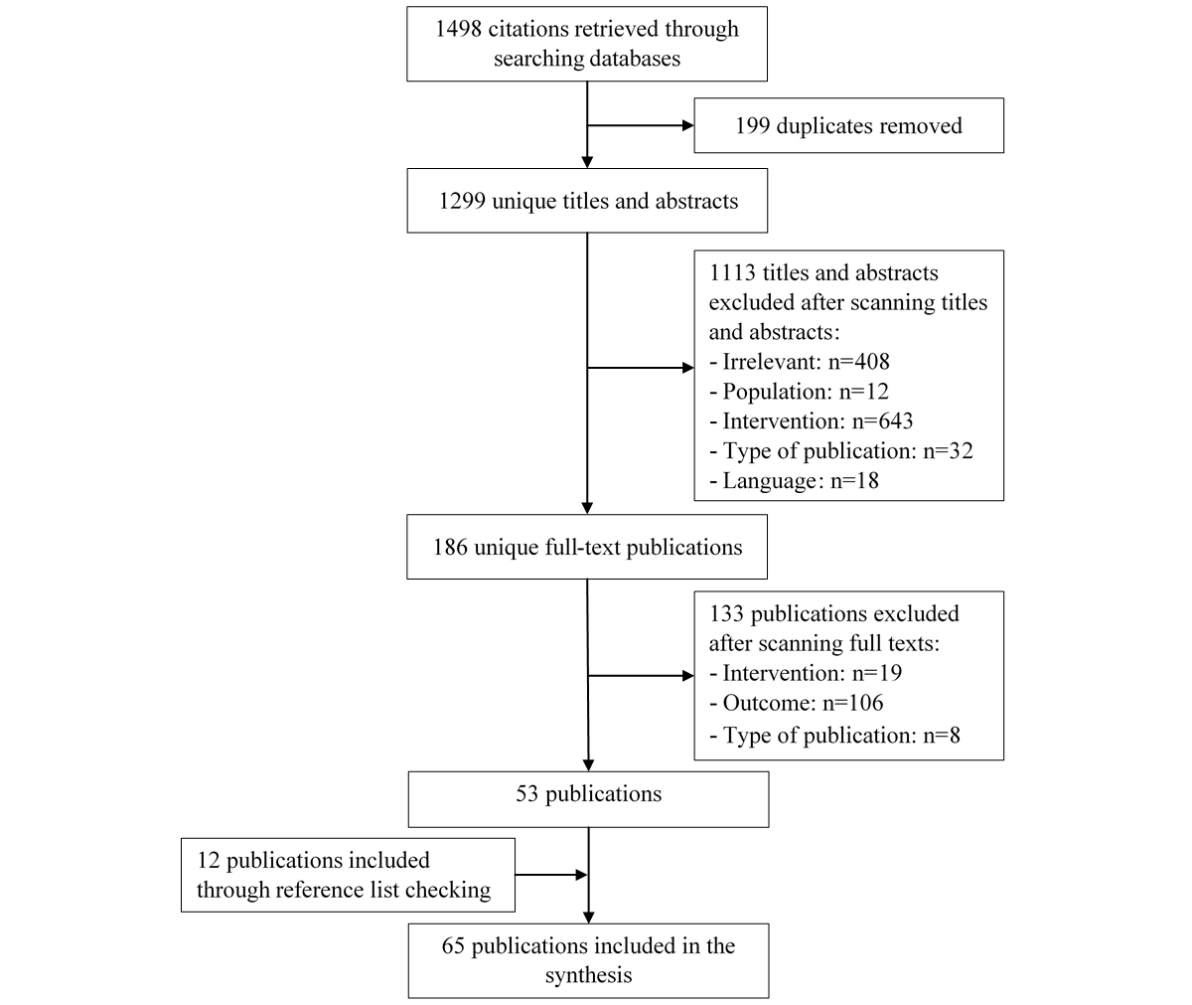
Flowchart of the study selection process.

By searching the 7 electronic databases, 1498 citations were retrieved. After removing 199 (13.3%) duplicates of these citations, 1299 (86.7%) titles and abstracts were screened. The screening process resulted in excluding 1113 (74.3%) titles and abstracts due to several reasons detailed in Figure 1. When we read the full text of the remaining 186 (12.4%) citations, a further 133 (8.9%) citations were excluded (Figure 1). In total, 12 studies were found by backward and forward reference checking. We included 65 studies in the review.

### Description of Included Studies

Characteristics of the included studies are detailed in Table 1. Cross-sectional survey was the most commonly used study design (n=41, 63%). About 57% (n=37) of the included studies were published as journal articles. Half of the studies (n=33, 51%) were conducted in the United States. Approximately 70% (n=45) of the studies were published between 2015 and 2019.

The sample size was reported in 61 studies, and 38 studies (62%) had 50 or fewer participants. In 44 studies, the age of participants was reported; the mean age of participants was 39 years, with a range of 13-79 years. Sex of participants was reported in 54 studies, where the mean percentage of males was 48.1%. Of the 62 studies that reported participants’ health conditions, 34 (54.8%) studies recruited participants from a clinical population (ie, those with health issues). Participants were recruited from clinical settings (n=30, 49.2%), community (n=20, 32.8%), and educational settings (n=18, 29.5%). Metadata and population characteristics of each included study are presented in Multimedia Appendix 3.

Chatbots were used for self-management (n=17, 26.2%), therapeutic purposes (n=12, 18.5%), counselling (n=12, 18.5%), education (n=10, 15.4%), screening (n=9, 13.8%), training (n=7, 10.8%), and diagnosing (n=3, 4.6%). Although chatbots were implemented in stand-alone software in about 62% (n=40) of studies, chatbots were implemented in web-based platforms in the remaining studies (n=25, 39%). Chatbot responses were generated based on predefined rules, machine learning approaches, or both methods (hybrid) in 82% (N=53), 17% (n=11), and 2% (n=1) of the included studies, respectively. In the majority of studies (n=58, 89%), chatbots led the dialogue. In about 62% (n=40) of studies, users interacted with chatbots only by typing in their utterances (texts). The most common modalities used by chatbots to interact with users were a combination of text, voice, and nonverbal language (ie, facial expression and body language; n=21, 32%), text only (n=20, 31%), and a combination of voice and nonverbal language (n=19, 29%). The most common disorders targeted by chatbots were any health condition (n=20, 31%) and depression (n=15, 23%). Multimedia Appendix 4 displays characteristics of the intervention in each included study.

**Table 1:** Characteristics of the included studies (N=65).

Parameters and characteristics				Studies, n (%)^a^
**Study metadata**				
	**Study design**			
		Survey	41 (63)	
		Quasi-experiment	11 (17)	
		Randomized controlled trial	13 (20)	
	**Type of publication**			
		Journal article	37 (57)	
		Conference proceeding	25 (39)	
		Thesis	3 (5)	
	**Country**			
		United States	33 (51)	
		France	5 (8)	
		Netherlands	3 (5)	
		Japan	3 (5)	
		Australia	3 (5)	
		Italy	2 (3)	
		Switzerland and Netherlands	2 (3)	
		Finland	1 (2)	
		Sweden	1 (2)	
		Turkey	1 (2)	
		United Kingdom	1 (2)	
		Switzerland & Germany	1 (2)	
		Mexico	1 (2)	
		Spain	1 (2)	
		Global population	1 (2)	
		Romania, Spain and Scotland	1 (2)	
		Philippines	1 (2)	
		Switzerland	1 (2)	
		New Zealand	1 (2)	
		Spain and New Zealand	1 (2)	
		South Africa	1 (2)	
	**Year of publication**			
		Before 2010	3 (5)	
		2010-2014	17 (26)	
		2015-2019	45 (70)	
**Population** **characteristics**				
	**Sample size^b^**			
		≤50	38 (62)	
		51-100	11 (18)	
		101-200	9 (15)	
		>200	3 (5)	
	**Age (years)^c^**			
		Mean (range)	39 (13-79)	
	**Sex (%)^e^**			
		Male	48.1	
	**Health condition^f^**			
		Clinical sample	34 (55)	
		Nonclinical sample	28 (45)	
	**Setting^g,h^**			
		Clinical	30 (50)	
		Community	20 (33)	
		Educational	18 (30)	
**Intervention characteristics**				
	**Purpose^i^**			
		Self-management	17 (26)	
		Therapy	12 (19)	
		Counselling	12 (19)	
		Education	10 (15)	
		Screening	9 (14)	
		Training	7 (11)	
		Diagnosing	3 (5)	
	**Platform**			
		Stand-alone software	40 (62)	
		Web-based	25 (39)	
	**Response generation**			
		Rule-based	53 (82)	
		Artificial intelligence	11 (17)	
		Hybrid	1 (2)	
	**Dialogue initiative**			
		Chatbot	58 (89)	
		Users	4 (6)	
		Both	3 (5)	
	**Input modality**			
		Text	40 (62)	
		Voice	9 (14)	
		Voice and nonverbal	8 (12)	
		Text and voice	6 (9)	
		Text and nonverbal	2 (3)	
	**Output modality**			
		Text, voice and nonverbal	21 (32)	
		Text	20 (31)	
		Voice and nonverbal	19 (29)	
		Text & voice	4 (6)	
		Voice	1 (2)	
	**Targeted disorders^j^**			
		Any health condition	20 (31)	
		Depression	15 (23)	
		Autism	5 (8)	
		Anxiety	5 (8)	
		Substance use disorder	5 (8)	
		Posttraumatic stress disorder	5 (8)	
		Mental disorders	3 (5)	
		Sexually transmitted diseases	3 (5)	
		Sleep disorders	2 (3)	
		Diabetes	2 (3)	
		Alzheimer	1 (2)	
		Asthma	1 (2)	
		Cervical cancer	1 (2)	
		Dementia	1 (2)	
		Schizophrenia	1 (2)	
		Stress	1 (2)	
		Genetic variants	1 (2)	
		Cognitive impairment	1 (2)	
		Atrial fibrillation	1 (2)	


^a^Percentages were rounded and may not sum to 100.

^b^Sample size was reported in 61 studies.

^c^Mean age was reported in 44 studies.

^d^N/A: not applicable.

^e^Sex was reported in 54 studies.

^f^Sample type was reported in 62 studies.

^g^Setting was reported in 61 studies.

^h^Numbers do not add up as several chatbots focused on more than one health condition.

^i^Numbers do not add up as several chatbots have more than one purpose.

^j^Numbers do not add up as several chatbots target more than one health condition.

### Results of Studies

#### Overview

The included studies evaluated chatbots using many technical metrics, which were categorized into 4 main groups: metrics related to chatbots as a whole (global metrics), metrics related to response generation, metrics related to response understanding, and metrics related to esthetics. More details about metrics are presented in the following sections.

#### Global Metrics

The included studies evaluated chatbots as a whole using the following metrics: usability, classifier performance, speed, technical issues, intelligence, task completion rate, dialogue efficiency, dialogue handling, context awareness, and error management.

Usability of chatbots was assessed in 37 (56.9%) studies [17-53]. Usability was evaluated using a single question in a self-administrated questionnaire [17,20-25,29-31,33,34,36,37,40,42,44,45,47-51,53], multiple questions in a self-administrated questionnaire [28,41,43], a specific questionnaire (eg, system usability scale [SUS] questionnaire) [18,26,27,32,35,38,39,46,52], or observation [19].

Classifier performance of chatbots was evaluated in 8 (12.3%) studies [54-61]. Many metrics were used to measure the classifier performance, namely: area under curve [54,55,60,61], accuracy [56-58,61], sensitivity [55,57,59,60], specificity [55,57,59,60], positive predictive value [55,57,60], and negative predictive value [55,60]. The speed of chatbots was examined in 4 studies [29,35,53,62]. The speed was evaluated using a single question in a self-administrated questionnaire [29,35], multiple questions in a self-administrated questionnaire [53], and interviews [62].

Technical issues (eg, errors/glitches) in chatbots were examined in 4 studies (6.2%) [7,36,51,63]. Technical issues were assessed through interviews [7,51,63], a single question in a self-administrated questionnaire [36], and checking staff logs [51]. In addition, 3 studies assessed the intelligence of chatbots using either multiple questions in a self-administrated questionnaire [41,64] or a single question in a self-administrated questionnaire [27]. In 3 studies, the task completion rate was examined by checking the conversation logs [38,53,65].

Of the reviewed studies, 2 (3.1%) studies examined chatbot flexibility in dialogue handling (eg, its ability to maintain a conversation and deal with users’ generic questions or responses that require more, less, or different information than was requested) using interviews [27] and multiple questions in a self-administrated questionnaire [38]. Dialogue efficiency of chatbots, which refers to the number of dialogue steps required to finish a task, was assessed in 1 study by reviewing transcribed conversation logs [38]. The same study examined the chatbot’s context awareness (ie, its ability to utilize contextual knowledge to appropriately respond to users) using multiple questions in a self-administrated questionnaire [38]. Error management, which refers to a chatbot’s ability to detect and understand misspelled words in users’ replies (eg, “anious” instead of anxious), was examined in only 1 study [27].

#### Metrics Related to Response Generation

The following metrics were utilized by the included studies to evaluate response generation by chatbots: appropriateness of responses, comprehensibility, realism, speed of response, empathy, repetitiveness, clarity of speech, and linguistic accuracy.

Of the reviewed studies, 15 (23.1%) examined the appropriateness and adequacy of verbal [18,19,27,28,31,34,38,39,51,58,66-69] and nonverbal responses of chatbots [32]. Appropriateness of responses was assessed using interviews [18,19,31,34,51,66,68], a single question in self-administrated questionnaire [27,32,39,67], conversation logs [38,58,69], and multiple questions in self-administrated questionnaire [28].

Comprehensibility of responses, which refers to the degree to which a chatbot generates responses understandable by users, was evaluated by 14 (21.5%) studies [20,23,31,34,36,39,42,44,51,52,59,60,63,69]. Comprehensibility of responses was evaluated using a single question in a self-administrated questionnaire [20,23,31,36,39,42,44,52,59,60,63,69] and interviews [34,51].

In total, 14 (21.5%) studies assessed how human-like chatbots are (realism) [17,18,21,34,39,41,46,50,63,66,68,70-72]. Realism of chatbots was examined in terms of verbal responses only [17,21,34,39,46,63,68,70], nonverbal responses only [66], or both verbal and nonverbal responses [18,41,50,71,72]. The included studies evaluated realism using a single question in a self-administrated questionnaire [17,18,21,39,46,50,63,70], multiple questions in a self-administrated questionnaire [41,72], and interviews [18,34,66,68,71].

Altogether, 11 (16.9%) studies assessed the speed of a chatbot’s responses [18,19,28,30,34,36,38,68-70,73]. The speed of responses was examined using a single question in a self-administrated questionnaire [18,30,36,69,70,73], interviews [19,34,68], multiple questions in a self-administrated questionnaire [53], and system logs [38]. Empathy of a chatbot, which refers to its ability to show empathy to users, was examined in 10 studies [7,35,41,42,64,66,67,71,73,74]. Those studies evaluated empathy using a single question in a self-administrated questionnaire [7,35,41,42,67,71,73], interviews [66,71], and multiple questions in a self-administrated questionnaire [64].

Repetitiveness of a chatbot’s responses was examined in 9 (13.8%) studies [7,20,27,53,57,66,73,75,76]. Repetitiveness of responses was evaluated using a single question in a self-administrated questionnaire [7,20,27,53,57,73] and interviews [66,75,76]. We found that 6 (9.2%) studies evaluated clarity or quality of speech using either interviews [51,62,75] or a single question in a self-administrated questionnaire [27,69,77]. Linguistic accuracy of a chatbot’s responses was evaluated in 2 (3.7%) studies using a single question in a self-administrated questionnaire [31,35].

#### Metrics Related to Response Understanding

The included studies evaluated chatbot understanding of users’ responses using the following metrics: understanding as assessed by users, word error rate, concept error rate, and attention estimator errors.

Chatbot understanding, which refers to a chatbot’s ability to adequately understand the verbal and nonverbal responses of users, was assessed by 20 (30.8%) studies [7,18,20,23,27,32,33,36,39,41,42,53,57,59,63,64,68,73,78,79]. Of those studies, 2 studies assessed understanding of both verbal and nonverbal responses [18,79], 1 study assessed understanding of nonverbal responses only [32], and the remaining studies assessed understanding of verbal responses only. The understanding of responses was evaluated using multiple questions in a self-administrated questionnaire in 4 studies [42,64,78,79], interviews in 2 studies [18,68], and a single question in a self-administrated questionnaire in the remaining studies.

Word error rate, which assesses the performance of speech recognition in chatbots, was examined in 2 (3.7%) studies using conversational logs [65,69]. Concept error rate, which depends on the correct recognition of the semantic result of a user utterance, was evaluated in 1 study by checking conversational logs [65]. Attention estimation, which refers to a chatbot’s ability to determine whether the user is gazing toward the screen or away from it, was examined in 1 study by checking conversational logs [69].

#### Metrics Related to Esthetics

The included studies evaluated the esthetics of chatbots using the following metrics: appearance of the virtual agent, background color and content, font type and size, button color, shape, icon, and background color contrast.

In total, 5 (7.7%) studies assessed the appearance of the virtual agent using a single question in a self-administrated questionnaire [69,77,80], interviews [51], and focus groups [45]. In addition, 1 (1.5%) study evaluated background color, color contrast, and content; font type and size; and button color, shape, and icon using a survey [80].

## Discussion

### Principal Findings

It became clear that there is currently no standard method in use to evaluate health chatbots. Most aspects are studied using self-administered questionnaires or user interviews. Common metrics are response speed, word error rate, concept error rate, dialogue efficiency, attention estimation, and task completion. Various studies assessed different aspects of chatbots, complicating direct comparison. Although some of this variation may be due to the individual characteristics of chatbot implementations and their distinct use cases, it is difficult to see why metrics such as appropriateness of responses, comprehensibility, realism, speed of response, empathy and repetitiveness are each only applicable to a small percentage of cases. Further, objective quantitative metrics (eg, those based on log reviews) were comparatively rarely used in the reported studies. We thus suggest continuing research and development toward an evaluation framework for technical metrics with recommendations for specific circumstances for their inclusion in chatbot studies.

Jadeja et al [81] introduced 4 dimensions for chatbot evaluations: the information retrieval (IR) perspective, the user experience (UX) perspective, the linguistic perspective, and the AI (human-likeness) perspective. In earlier work [14], we adapted and broadened this categorization, modifying the IR perspective to a task-oriented perspective since health chatbots are not necessarily designed only to retrieve information; additionally, we included system quality and health care quality perspectives. Excluding the health care quality perspective, which is outside the definition of technical metrics, the findings of this scoping review show that all these dimensions are indeed represented in health chatbot evaluations. Rather, the issue appears to be the inconsistency in what is measured and how, along with the skew toward self-reporting and the UX perspective. Additional work is still required to come up with standard metrics and corresponding assessment tools specifically addressing quality in health chatbots.

We found usability to be the most commonly assessed aspect of health chatbots. The system usability scale (SUS [82,83]) is a well-established usability instrument that we observed was used repeatedly, although it was not used in the majority of the studies assessing usability; in many cases, a single survey question was used instead. The SUS is nonproprietary, technology-agnostic, and designed to support comparison across products [82]. As such, global assessment of the user experience of health chatbots could be enhanced in quality and comparability by researchers standardizing on inclusion of the SUS in their evaluations. However, studies by Holmes et al [84] showed that conventional methods for assessing usability and user experience may not be as accurate when applied to health chatbots. As such, there remains research to be done toward appropriate metrics for health chatbots.

Conversational-turns per session (CPS) has been suggested as a success metric for social chatbots as exemplified by XiaoIce [85]. Although the aims for health chatbots are not identical to those of social chatbots, if CPS gains acceptance as a standard measure in the social chatbot domain, it would make it a leading candidate for a standard measure to include in health chatbot evaluations to assess their social engagement dimension. An alternative or supplementary measure related to the social dimension would be to have users score the chatbot on empathy; however, CPS has the advantage of being an objective and quantitative measure. Other objective and quantitative measures such as interaction time or time on task could be alternatives to CPS, but might be less representative of engagement than CPS if for instance the user was multitasking chatbot interaction with other tasks. Beyond social engagement, task completion (often assessed by analyzing conversation logs) is another promising global measure.

A further area for standardization would be in the quality of responses. We observed response generation to be widely measured but in very diverse ways. Emergence of standard measures for response generation and understanding would greatly advance the comparability of studies. Development of validated instruments in this area would be a useful contribution to chatbot research.

We commend the inclusion of classifier performance in health chatbot studies where this is applicable and practical to assess. It could be less meaningful to compare raw performance (eg, as area under the curve) across domains due to differences in difficulty; ideally, chatbot performance would be compared to the performance of a human expert for the task at hand. Further, we perceive the opportunity for a progression of performance measures in health chatbot studies as a given product gains maturity. Good early-stage metrics would be those that assess response quality and response understanding to establish that the product is working well. Subsequent experiments can advance the assessment of self-reported usability and metrics of social engagement. Where applicable, classifier performance can round out technical performance evaluation to establish whether trials to assess clinical outcomes are warranted.

### Strengths and Limitations

#### Strengths

This study is the first review that summaries the technical metrics used by previous studies to evaluate health care chatbots. This helps readers explore how chatbots were evaluated in health care. Given that this review was executed and reported in line with PRISMA-ScR guidelines [1], it could be considered a high-quality review.

To retrieve as many relevant studies as possible, the most commonly used databases in the fields of health and information technology were searched. Further, we searched Google Scholar and conducted backward and forward reference list checking to retrieve gray literature and minimize the risk of publication bias.

As two reviewers independently selected the studies and extracted the data, the selection bias in this review was minimal. This review can be considered a comprehensive review given that we did not apply restrictions regarding the study design, study setting, year of publication, and country of publication.

Laranjo et al [86] reviewed the characteristics, current applications and evaluation measures of health chatbots. In contrast to our work, they did not solely concentrate on the technical metrics used for chatbot evaluations. The metrics they reviewed included task completion or word accuracy. In contrast to Laranjo et al [86], who included only 17 papers reporting on 14 different conversational agents, our work is more comprehensive as it included 65 publications. Further, we had a different research question in mind while conducting the review.

#### Limitations

This review focused on chatbots that are aimed at delivering health care services to patients and work on stand-alone software and web browsers; it excluded chatbots that used robotics, serious games, SMS text messaging, Wizard-of-Oz, and telephones. Thus, this review did not include many technical metrics used to evaluate chatbots for other users (eg, physicians, nurses, and caregivers), in other fields (eg, business and education), or with alternative modes of delivery (eg, SMS text messaging, Wizard-of-Oz, and telephones). The abovementioned restrictions were applied by previous reviews about chatbots as these features are not part of ordinary chatbots [87-90].

Due to practical constraints, we could not search interdisciplinary databases (eg, Web of Science and ProQuest), conduct a manual search, or contact experts. Further, the search in this review was restricted to English-language studies. Accordingly, it is likely that this review missed some studies.

### Conclusion

From this review, we perceive the need for health chatbot evaluators to consider measurements across a range of aspects in any given study or study series, including usability, social experience, response quality, and, where applicable, classifier performance. The establishment of standard measures would greatly enhance comparability across studies with the SUS and CPS as leading candidates for usability and social experience, respectively. It would be ideal to develop guidelines for health chatbot evaluators indicating what should be measured and at what stages in product development. Development of validated measurement instruments in this domain is sparse and such instruments would benefit the field, especially for response quality metrics.

## Appendix

Multimedia Appendix 1 Search string.

Multimedia Appendix 2 Data extraction form.

Multimedia Appendix 3 Metadata and population characteristics of each included study.

Multimedia Appendix 4 Characteristics of the intervention in each included study.

## Abbreviations

ACM: Association for Computing Machinery
AI: artificial intelligence
CINAHL: Cumulative Index of Nursing and Allied Health Literature
CPS: conversational-turns per session
eHealth: electronic health
EMBASE: Excerpta Medica Database
IEEE: Institute of Electrical and Electronics Engineers
IR: information retrieval
PRISMA-ScR: Preferred Reporting Items for Systematic Reviews and Meta-Analyses-Extension for Scoping Reviews
UX: user experience
