# Supplementary material for: Technical Metrics Used to Evaluate Health Care Chatbots: Scoping Review
Source: J Med Internet Res. 2020 Jun 5;22(6):e18301. doi: 10.2196/18301 (PMC7305563; doi:10.2196/18301)
Supplement: Multimedia Appendix 1 [file jmir_v22i6e18301_app1.docx]

Database(s): **Embase**1974 to 2019 November 01 via Ovid
Search Strategy:

| **#** | **Searches** | **Results** |
| --- | --- | --- |
| 1 | conversational agent*.tw. | 89 |
| 2 | conversational bot*.tw. | 1 |
| 3 | conversational system*.tw. | 12 |
| 4 | chatbot*.tw. | 76 |
| 5 | chat bot*.tw. | 23 |
| 6 | chatterbot*.tw. | 0 |
| 7 | chatter bot*.tw. | 0 |
| 8 | chat-bot*.tw. | 23 |
| 9 | smartbot*.tw. | 2 |
| 10 | smart-bot*.tw. | 9 |
| 11 | smart bot*.tw. | 9 |
| 12 | relational agent*.tw. | 13 |
| 13 | dialog system.tw. | 16 |
| 14 | dialog agent.tw. | 0 |
| 15 | agent-based system.tw. | 26 |
| 16 | 1 or 2 or 3 or 4 or 5 or 6 or 7 or 8 or 9 or 10 or 11 or 12 or 13 or 14 or 15 | 251 |
| 17 | limit 16 to english language | 245 |
| 18 | limit 17 to exclude medline journals | 27 |

Database(s): **MEDLINE(R)**via EBSCO

| **#** | **Searches** | **Results** |
| --- | --- | --- |
| S18 | limit 16 to English language | 597 |
| S17 | S1 OR S2 OR S3 OR S4 OR S5 OR S6 OR S7 OR S8 OR S9 OR S10 OR S11 OR S12 OR S13 OR S14 OR S15 OR S16 OR S16 | 604 |
| S16 | AB "agent-based system*" | 28 |
| S15 | AB "dialog agent*" | 1 |
| S14 | AB "dialog system*" | 17 |
| S13 | AB "relational agent*" | 13 |
| S12 | AB "smart-bot*" | 4 |
| S11 | AB "smart bot*" | 4 |
| S10 | AB "smartbot*" | 1 |
| S9 | AB "chat-bot*" | 14 |
| S8 | AB "chatter-bot*" | 0 |
| S7 | AB "chatter bot*" | 0 |
| S6 | AB chatterbot* | 0 |
| S5 | AB chat bot* | 423 |
| S4 | AB chatbot* | 52 |
| S3 | AB "conversational system*" | 7 |
| S2 | AB "conversational bot*" | 1 |
| S1 | AB "conversational agent*" | 79 |

Database(s): **CINHAL** via EBSCO

| **#** | **Query** | **Results** |
| --- | --- | --- |
| S18 | limit 16 to English language | 153 |
| S17 | S1 OR S2 OR S3 OR S4 OR S5 OR S6 OR S7 OR S8 OR S9 OR S10 OR S11 OR S12 OR S13 OR S14 OR S15 OR S16 OR S17 | 153 |
| S16 | AB "agent-based system*" | 4 |
| S15 | AB "dialog agent*" | 0 |
| S14 | AB "dialog system*" | 4 |
| S13 | AB "relational agent*" | 5 |
| S12 | AB "smart-bot*" | 0 |
| S11 | AB "smart bot*" | 0 |
| S10 | AB "smartbot*" | 1 |
| S9 | AB "chat-bot*" | 6 |
| S8 | AB "chatter-bot*" | 0 |
| S7 | AB "chatter bot*" | 0 |
| S6 | AB chatterbot* | 0 |
| S5 | AB chat bot* | 29 |
| S4 | AB chatbot* | 73 |
| S3 | AB "conversational system*" | 2 |
| S2 | AB "conversational bot*" | 0 |
| S1 | AB "conversational agent*" | 50 |

Database(s): **PsycINFO via OVID**1806 to October Week 4 2019
Search Strategy:

| **#** | **Searches** | **Results** |
| --- | --- | --- |
| 1 | conversational agent*.tw. | 191 |
| 2 | conversational bot*.tw. | 1 |
| 3 | conversational system*.tw. | 18 |
| 4 | chatbot*.tw. | 51 |
| 5 | chat bot*.tw. | 14 |
| 6 | chatterbot*.tw. | 8 |
| 7 | chatter bot*.tw. | 1 |
| 8 | chat-bot*.tw. | 14 |
| 9 | smartbot*.tw. | 1 |
| 10 | smart-bot*.tw. | 1 |
| 11 | smart bot*.tw. | 1 |
| 12 | relational agent*.tw. | 22 |
| 13 | dialog system.tw. | 48 |
| 14 | dialog agent.tw. | 4 |
| 15 | agent-based system.tw. | 17 |
| 16 | 1 or 2 or 3 or 4 or 5 or 6 or 7 or 8 or 9 or 10 or 11 or 12 or 13 or 14 or 15 | 346 |
| 17 | limit 16 to english language | 324 |

| **Databases** | **Search strings** | **Hit** |
| --- | --- | --- |
| **ACM digital Library** | (("conversational agent*" OR "conversational bot" OR "conversational bots" OR "conversational system*" "chatbot*" OR "chat bot" OR "chat bots" OR "chat-bot" OR "chat-bots" OR "smartbot" OR "smartbots" OR "smart bot" OR "smart bot" OR "smart-bot" OR "smart-bots" OR "relational agent" OR "relational agents" OR "agent based system" OR "agent based systems") AND (+Health Disorder* Disease* well-being illness medical medicine patient patients) | 68 |
| **IEEE Xplore** | (("Abstract":"conversational agent*" OR "conversational bot" OR "conversational bots" OR "conversational system*" OR "chatbot*" OR "chat bot" OR "chat bots" OR "chat-bot" OR "chat-bots" OR "smartbot" OR "smartbots" OR "smart bot" OR "smart bot" OR "smart-bot" OR "smart-bots" OR "relational agent" OR "relational agents" OR "agent based system" OR "agent based systems") AND ("Full Text Only":Health OR Disorder* OR Disease* OR well-being OR illness OR medical OR medicine OR patient OR patients)) | 229 |
| **Google Scholar** | ((“conversational agent*” OR “conversational bot*” OR "conversational system*" OR “conversational interface*” OR “chatbot*” OR “chat bot*” OR “chat-bot*”) AND (Health OR Disorder* OR Disease* OR patient* OR illness OR medical) | 100 |
