# Supplementary material for: Technical Metrics Used to Evaluate Health Care Chatbots: Scoping Review
Source: J Med Internet Res. 2020 Jun 5;22(6):e18301. doi: 10.2196/18301 (PMC7305563; doi:10.2196/18301)
Supplement: Multimedia Appendix 3 [file jmir_v22i6e18301_app3.docx]

| Author (year)^ID^ | Country | Publication type | Study design | Sample size | Mean age | Sex (male) | Sample type | Recruitment Setting |
| --- | --- | --- | --- | --- | --- | --- | --- | --- |
| Abdullah (2018)^16^ | USA | Journal | Survey | 6 | 56.3 | 67% | Clinical | Clinical |
| Ali (2018)^17^ | USA | Conference proc. | Survey | 9 | - | - | Clinical | Clinical |
| Amato (2017)^53^ | Italy | Conference proc. | Quasi-experiment | - | - | - | Clinical | Clinical |
| Auriacombe (2018)^54^ | France | Journal | Survey | 139 | 43 | 49% | Clinical | Clinical |
| Beiley (2019)^18^ | USA | Thesis | Survey | 3 | 21 | 33.3% | Non-clinical | Educational |
| Bickmore (2003)^74^ | USA | Thesis | RCT | 91 | 24.9 | 43% | Non-clinical | Educational |
| Bickmore (2005)^19^ | USA | Conference proc. | Survey | 10 | 72.8 | 0% | Non-clinical | Clinical |
| Bickmore (2009)^20^ | USA | Conference proc. | Survey | 19 | 55.42 | 47% | Clinical | Clinical |
| Bickmore (2010a)^21^ | USA | Journal | Survey | 131 | 48 | 54% | Clinical | Clinical |
| Bickmore (2010b)^22^ | USA | Journal | RCT | 176 | 54 | 48.3% | Clinical | Clinical |
| Bickmore (2010c)^23^ | USA | Journal | Survey | 20 | - | 33% | Clinical | Clinical |
| Bickmore (2013)^24^ | USA | Journal | RCT | 122 | 33 | 39.3% | Non-clinical | Community |
| Bresó (2016)^25^ | Spain & Mexico | Journal | Survey | 60 | - | 55% | Non-clinical | Educational |
| Burton (2016)^65^ | Romania, Spain & Scotland | Journal | Survey | 13 | 35.3 | 23% | Clinical | Clinical |
| Cameron (2018)^26^ | UK | Conference proc. | Survey | 7 | - | 29% | Non-clinical | Clinical |
| Comendador (2015)^27^ | Philippines | Journal | Survey | 18 | - | - | Non-clinical | Clinical & Educational |
| Crutzen (2011)^28^ | Netherlands | Journal | Survey | 929 | 15 | 36% | Non-clinical | Community |
| Demirci (2018)^29^ | Turkey | Thesis | Survey | 16 | 28 | 56% | Non-clinical | Educational |
| Denecke (2018)^30^ | Switzerland & Germany | Journal | Survey | 22 | 39 | 59% | Non-clinical | Community & Educational |
| DeVault (2014)^31^ | USA | Conference proc. | Quasi-experiment | 351 | 42.6 | 62% | Non-clinical | Community |
| Dworkin (2019)^32^ | USA | Journal | Survey | 43 | 29 | 100% | Clinical | Clinical and Community |
| Elmasri (2016)^33^ | Australia | Conference proc. | Survey | 17 | 18-25 | 58.8% | Non-clinical | Educational |
| Fadhil (2013)^34^ | Italy | Conference proc. | Survey | 43 | 29.8 | 60.4% | Non-clinical | Community |
| Fitzpatrick (2017)^7^ | USA | Journal | RCT | 70 | 22.2 | 33% | Clinical | Community |
| Fulmer (2018)^72^ | USA | Journal | Survey | 50 | 23.1 | 28% | Non-clinical | Educational |
| Gardiner (2017)^61^ | USA | Journal | RCT | 61 | 35 | 0% | Clinical | Clinical |
| Ghosh (2018)^55^ | Australia | Conference proc. | Quasi-experiment | - | - | - | - | - |
| Griol (2015)^35^ | USA | Journal | Survey | 25 | 58.6 | 48% | Clinical | - |
| Hanke (2016)^36^ | Switzerland & Netherlands | Conference proc. | Survey | 18 | 73.7 | 27.7% | Clinical | community |
| Hess (2019)^37^ | Switzerland & Netherlands | Conference proc. | Quasi-experiment | - | - | - | - | - |
| Inkster (2018)^56^ | Global population | Journal | Survey | 129 | - | - | Non-clinical | Community |
| Kadariya (2019)^38^ | USA | Conference proc. | Survey | 16 | - | - | Non-clinical | Clinical & Educational |
| Kang (2018)^69^ | USA | Conference proc. | Quasi-experiment | 132 | 38.94 | 46.2% | Non-clinical | Community |
| Kowatsch (2017)^39^ | Switzerland | Conference proc. | Survey | 11 | 12.6 | 72.7% | Clinical | Clinical |
| Lisetti (2013)^40^ | USA | Conference proc. | Quasi-experiment | 81 | - | 63% | Non-clinical | Educational |
| Liu (2018)^63^ | USA | Journal | Quasi-experiment | 246 | 31.5 | 31.3% | Non-clinical | Community & Educational |
| Ly (2017)^75^ | Sweden | Journal | Survey | 9 | 28.8 | 44% | Non-clinical | Community & Educational |
| Magnani (2017)^41^ | USA | Journal | Survey | 31 | 68 | 61% | Clinical | Clinical |
| Martínez-Miranda (2014)^73^ | Spain | Conference proc. | Survey | 8 | - | 40% | Clinical | Community |
| Martínez-Miranda (2019)^76^ | Mexico | Journal | Survey | 18 | 31.5 | 38.9% | Clinical | clinical |
| Micoulaud (2016)^42^ | France | Conference proc. | Quasi-experiment | 178 | 46.5 | 42.7% | Clinical | Clinical |
| Milne (2010)^43^ | Australia | Conference proc. | Survey | 14 | 10 | - | Clinical | Clinical |
| Ni (2017)^57^ | New Zealand | Conference proc. | Quasi-experiment | - | - | - | - | - |
| Olafsson (2019)^70^ | USA | Conference proc. | RCT | 39 | 24.6 | 45% | Non-clinical | Community |
| Philip (2014)^58^ | France | Journal | Quasi-experiment | 62 | 42.2 | 41.9% | Clinical | Clinical & Community |
| Philip (2017)^59^ | France | Journal | RCT | 187 | 46.5 | 42.5% | Clinical | Clinical |
| Pinto (2015)^77^ | USA | Journal | RCT | 28 | 22 | 33% | Clinical | Community |
| Razavi (2016)^62^ | USA | Conference proc. | Survey | 5 | - | 80% | Clinical | - |
| Schmidlen (2019)^44^ | USA | Journal | Survey | 62 | - | 32% | Clinical | clinical |
| Schroeder (2018)^45^ | USA | Conference proc. | Survey | 73 | 37.3 | 9.5% | Clinical | clinical |
| Smith (2014a)^46^ | USA | Journal | RCT | 37 | 48.2 | 48.6% | Clinical | Clinical |
| Smith (2014b)^47^ | USA | Journal | RCT | 26 | 24.2 | 77% | Clinical | Clinical, Educational & Community |
| Smith (2015)^48^ | USA | Journal | RCT | 33 | 51 | 97% | Clinical | Clinical |
| Swartout (2013)^66^ | USA | Journal | Survey | 111 | 41 | 79.3% | Non-clinical | Community |
| Tanaka (2015)^49^ | Japan | Conference proc. | Survey | 10 | - | 60% | Non-clinical | Educational |
| Tanaka (2017)^60^ | Japan | Journal | Quasi-experiment | 29 | 75.1 | 86.2% | Clinical | Clinical & Educational |
| Thompson (2019)^50^ | USA | Journal | Survey | 52 | - | 15.4% | Clinical | Clinical |
| Tielman (2017a)^51^ | Netherlands | Journal | Survey | 4 | - | 50% | Clinical | Clinical |
| Tielman (2017b)^71^ | Netherlands | Journal | Survey | 46 | 22.9 | 82.6% | Non-clinical | Educational |
| Turunen (2011)^64^ | Finland | Journal | Survey | 28 | - | - | Non-clinical | Community |
| van Heerden (2017)^67^ | South Africa | Conference proc. | Survey | 10 | 30.2 | 50% | Non-clinical | Community |
| Wargnier (2018)^68^ | France | Journal | Survey | 14 | 78.8 | 21.4% | Clinical | Clinical |
| Wu (2014)^79^ | USA | Conference proc. | Survey | 26 | - | - | Clinical | Clinical |
| Yasavur (2014)^52^ | USA | Journal | Survey | 89 | - | 69.7% | Non-clinical | Educational |
| Yokotani (2018)^78^ | Japan | Journal | RCT | 55 | 22.92 | 45.5% | Non-clinical | Educational |
