# Supplementary material for: Technical Metrics Used to Evaluate Health Care Chatbots: Scoping Review
Source: J Med Internet Res. 2020 Jun 5;22(6):e18301. doi: 10.2196/18301 (PMC7305563; doi:10.2196/18301)
Supplement: Multimedia Appendix 4 [file jmir_v22i6e18301_app4.docx]

| Author (year)^ID^ | Chatbot name | Purpose | Platform | Response generation | Dialogue initiative | Input modality | Output modality | Targeted Disorder |
| --- | --- | --- | --- | --- | --- | --- | --- | --- |
| Abdullah (2018)^16^ | ECA-Q | Therapy | Software | Rule-based | System | Voice | Voice | Substance use disorder |
| Ali (2018)^17^ | LISSA | Training | Web-based | Rule-based | System | Voice & Non-verbal | Text, Voice & Non-verbal | Autism |
| Amato (2017)^53^ | HOLMeS | Counseling | Web-based | Artificial intelligence | System | Text | Text | Any health condition |
| Auriacombe (2018)^54^ | Jeanne | Screening | Software | Rule-based | System | Voice | Voice & Non-verbal | Substance use disorder |
| Beiley (2019)^18^ | Mikebot | Self-management | Software | Rule-based | System | Text | Text | Depression |
| Bickmore (2003)^74^ | Laura | Self-management | Software | Rule-based | System | Text | Text, Voice & Non-verbal | Any health condition |
| Bickmore (2005)^19^ | Laura | Self-management | Software | Rule-based | System | Text | Text, Voice & Non-verbal | Any health condition |
| Bickmore (2009)^20^ | Louise and Elizabeth | Education | Software | Rule-based | System | Text | Voice & Non-verbal | Any health condition |
| Bickmore (2010a)^21^ | Elizabeth | Education | Software | Rule-based | System | Text | Voice & Non-verbal | Depression |
| Bickmore (2010b)^22^ | - | Education | Software | Rule-based | System | Text | Text, Voice & Non-verbal | Any health condition |
| Bickmore (2010c)^23^ | Laura | Self-management | Software | Rule-based | System | Text | Voice & Non-verbal | Schizophrenia |
| Bickmore (2013)^24^ | Karen | Counseling | Software | Rule-based | System | Text | Voice & Non-verbal | Any health condition |
| Bresó (2016)^25^ | PrevenDep | Screening & Therapy | Software | Rule-based | System | Text | Text, Voice & Non-verbal | Depression |
| Burton (2016)^65^ | Help4Mood | Self-management | Software | Rule-based | System | Text | Text, Voice & Non-verbal | Depression |
| Cameron (2018)^26^ | iHelpr | Self-management | Web-based | Rule-based | System | Text | Text & Voice | Depression, anxiety, stress, sleep, & self-esteem. |
| Comendador (2015)^27^ | Pharmabot | Counseling | Software | Rule-based | User | Text | Text | Any health condition |
| Crutzen (2011)^28^ | Bzz | Counseling | Web-based | Artificial intelligence | User | Text | Text | Any health condition |
| Demirci (2018)^29^ | Woebot | Therapy | Web-based | Rule-based | System | Text | Text | Depression & Anxiety |
| Denecke (2018)^30^ | Ana | Screening | Software | Rule-based | System | Text & Voice | Text & Voice | Any health condition |
| DeVault (2014)^31^ | SimSensei Kiosk | Screening | Software | Rule-based | System | Voice & Non-verbal | Voice & Non-verbal | Depession, anxiety & PTSD |
| Dworkin (2019)^32^ | My Personal Health Guide | Self-management | Software | Rule-based | System | Text | Text | Sexually transmitted diseases |
| Elmasri (2016)^33^ | - | Education & Screening | Software | Rule-based | System | Text | Text | Substance use disorder |
| Fadhil (2013)^34^ | Ollobot | Education & Self-management | Web-based | Artificial intelligence | User | Text | Text | Any health condition |
| Fitzpatrick (2017)^7^ | Woebot | Therapy | Web-based | Artificial intelligence | System | Text | Text | Depression and Anxiety |
| Fulmer (2018)^72^ | Tess | Therapy | Web-based | Artificial intelligence | System | Text | Text | Depression & Anxiety |
| Gardiner (2017)^61^ | Gabby | Education | Web-based | Rule-based | System | Voice & Non-verbal | Voice & Non-verbal | Any health condition |
| Ghosh (2018)^55^ | - | Screening | Web-based | Rule-based | System | Text | Text | any health condition |
| Griol (2015)^35^ | NA | Self-management | Web-based | Artificial intelligence | System | Text & Voice | Text & Voice | Alzheimer |
| Hanke (2016)^36^ | - | Self-management | Web-based | Artificial intelligence | System | Voice & Non-verbal | Voice & Non-verbal | Any health condition |
| Hess (2019)^37^ | eMMA | Self-management | Software | Rule-based | System | Text | Text | Any health condition |
| Inkster (2018)^56^ | Wysa | Therapy | Software | Artificial intelligence | System | Text | Text | Depression |
| Kadariya (2019)^38^ | kBOT | Self-management | Web-based | Rule-based | System | Text | Text & Voice | Asthma |
| Kang (2018)^69^ | Andy | Education | Web-based | Rule-based | System | Text | Text | Stress |
| Kowatsch (2017)^39^ | Anna or Lukas | Self-management | Software | Rule-based | System | Text | Text | Any health condition |
| Lisetti (2013)^40^ | Amy | Therapy | Web-based | Hybrid | System | Text & Non-verbal | Voice & Non-verbal | Substance use disorder |
| Liu (2018)^63^ | - | Counseling | Web-based | Rule-based | System | Text | Text | Sexually transmitted diseases |
| Ly (2017)^75^ | Shim | Therapy | Web-based | Rule-based | System | Text | Text | Any health condition |
| Magnani (2017)^41^ | Tanya | Counseling & Education | Software | Rule-based | System | Text | Voice & Non-verbal | Atrial Fibrillation |
| Martínez-Miranda (2014)^73^ | Help4Mood | Therapy | Software | Rule-based | System | Text | Text, Voice & Non-verbal | Depression |
| Martínez-Miranda (2019)^76^ | HelPath | Education & Screening | Software | Rule-based | System | Text | Text, Voice & Non-verbal | Any health condition |
| Micoulaud (2016)^42^ | - | Diagnosing | Software | Rule-based | System | Voice | Voice & Non-verbal | Depression |
| Milne (2010)^43^ | Thinking Head | Training | Software | Rule-based | System | Text & Non-verbal | Text, Voice & Non-verbal | Autism |
| Ni (2017)^57^ | Mandy | Diagnosing | Web-based | Rule-based | System | Text | Text | Any health condition |
| Olafsson (2019)^70^ | Emily and Katherine | Counseling | Software | Rule-based | System | Text | Voice & Non-verbal | Any health condition |
| Philip (2014)^58^ | - | Screening | Software | Rule-based | System | Voice | Voice & Non-verbal | Sleep disorder |
| Philip (2017)^59^ | - | Diagnosing | Software | Rule-based | System | Voice | Voice & Non-verbal | Depression |
| Pinto (2015)^77^ | eSMART-MH | Self-Management | Software | Rule-based | Both | Voice | Voice & Non-verbal | Depression |
| Razavi (2016)^62^ | LISSA | Training | Web-based | Rule-based | System | Voice & Non-verbal | Text, Voice & Non-verbal | Autism |
| Schmidlen (2019)^44^ | GIA | Counseling | Web-based | Rule-based | System | Text | Text | Genetic variants |
| Schroeder (2018)^45^ | Pocket Skills | Therapy | Web-based | Rule-based | System | Text | Text, Voice & Non-verbal | Mental disorders |
| Smith (2014a)^46^ | VR-JIT | Training | Software | Rule-based | System | Text & Voice | Text, Voice & Non-verbal | Mental disorders |
| Smith (2014b)^47^ | VR-JIT | Training | Software | Rule-based | System | Text & Voice | Text, Voice & Non-verbal | Autism |
| Smith (2015)^48^ | VR-JIT | Training | Software | Rule-based | System | Text & Voice | Text, Voice & Non-verbal | Posttraumatic stress disorder |
| Swartout (2013)^66^ | SimCoach | Counseling | Web-based | Artificial intelligence | Both | Text | Text, Voice & Non-verbal | Depression & Posttraumatic stress disorder |
| Tanaka (2015)^49^ | - | Training | Software | Rule-based | System | Voice & Non-verbal | Text, Voice & Non-verbal | Autism |
| Tanaka (2017)^60^ | - | Screening | Software | Rule-based | System | Voice & Non-verbal | Text, Voice & Non-verbal | Dementia |
| Thompson (2019)^50^ | Ashley | Self-management | Web-based | Rule-based | System | Text & Voice | Text, Voice & Non-verbal | Diabetes |
| Tielman (2017a)^51^ | 3MR_2 | Therapy | Software | Rule-based | System | Text | Text, Voice & Non-verbal | Posttraumatic stress disorder |
| Tielman (2017b)^71^ | 3MR | Therapy | Software | Rule-based | System | Text | Text, Voice & Non-verbal | Posttraumatic stress disorder |
| Turunen (2011)^64^ | - | Self-management | Web-based | Artificial intelligence | Both | Voice | Text, Voice & Non-verbal | Any health condition |
| van Heerden (2017)^67^ | Lwazi or Nolwazi | Counseling | Web-based | Rule-based | User | Text | Text | Sexually transmitted diseases |
| Wargnier (2018)^68^ | LOUISE | Self-management | Software | Rule-based | System | Voice & Non-verbal | Voice & Non-verbal | Cognitive impairment |
| Wu (2014)^79^ | - | Education | Software | Rule-based | System | Text | Voice & Non-verbal | Cervical cancer |
| Yasavur (2014)^52^ | - | Counseling | Software | Artificial intelligence | System | Voice | Voice & Non-verbal | Substance use disorder |
| Yokotani (2018)^78^ | - | Counseling | Software | Rule-based | System | Voice | Voice & Non-verbal | Mental disorders |
